# Supplementary material for: Comparison of DNA Extraction and Amplification Techniques for Use with Engorged Hard-Bodied Ticks
Source: Microorganisms. 2022 Jun 20;10(6):1254. doi: 10.3390/microorganisms10061254 (PMC9228219; doi:10.3390/microorganisms10061254)
Supplement: Supplementary file 1 [file microorganisms-10-01254-s001.zip › Supplemental Materials File S1_Protocol Handout copy.pdf]

Protocol Handouts

Tick Preparation

Store each engorged tick in 95% ethanol prior to processing

1. Remove from ethanol, dry, weigh and measure the length of each tick
2. Place the tick in a weigh boat or easily cleaned surface and cut into quarters by cutting both laterally and medially with a scalpel.
3. Place the tick contents into a sterile 1.5 ml microcentrifuge tube.
4. To prevent cross-contamination, clean scalpel and other tools in bleach in between each tick.

Phenol-Chloroform DNA Extraction

1. Add 200 µl of proteinase K (0.2 mg/mL) to prepared tick
2. Incubate for 16 hours at 55°C
3. Following incubation, vortex to disrupt any remaining solids
4. Centrifuged at 14,000 RPM for 10 minutes
5. Transfer the supernatant to a clean 1.5 ml microcentrifuge tube and add 400 µl of phenol:chloroform:isoamyl alcohol
6. Vortex to mix, centrifuge at 14,000 RPM for 2 minutes
7. Transfer the upper aqueous phase to a clean tube; Avoid any white precipitate between layers
8. Add 400 µl of phenol:chloroform:isoamyl alcohol and vortex to mix
9. Centrifuge at 14,000 RPM for 2 minutes
10. Transfer the upper aqueous phase to a clean tube; Avoid any white precipitate between layers
11. Add 400 µl of 3 M sodium acetate (pH 5.2) and 1200 µl of cold 100% ethanol to the clean tube; Invert tube to mix
12. Place tube in -20°C storage for 2 hours or 30 minutes at -70°C to precipitate DNA
13. Remove the tube from cold storage and centrifugate at 14,000 RPM for 10 minutes
14. Carefully pipette ethanol layer off the pellets of DNA
15. Add 500 µl of 70% ethanol to wash the DNA pellets
16. Centrifuge at 14,000 RPM and carefully remove the ethanol layer, being careful not to disrupt the DNA pellet
17. Allow all DNA pellets to air dry
18. Resuspend pellets in 50 µl of molecular water by mixing with pipette tip (do not pipette up and down)

Qiagen DNeasy Blood & Tissue Kit Extraction

1. Add 180 µl of Buffer ATL and 20 µl proteinase K (0.2 mg/ml) to tick
2. Incubate at 56°C overnight
3. Spin down samples at 14,000 RPM for 10 minutes and transfer supernatant to a new tube.
4. Add 200 µl of Buffer ATL to the sample, and vortex thoroughly
5. Add 200 µl of 100% ethanol, vortex again
6. Pipette mixture into a spin column
7. Centrifuge at 8,000 RPM for 1 minute, discard flow through
8. Add 500 µl of Buffer AW1, centrifuge for 1 min at 8,000 RPM, discard flow through
9. Add 500 µl of Buffer AW2, centrifuge for 3 minutes at 14,000 RPM
10. Transfer the spin column to a clean tube
11. Add 200 µl of Buffer AE to the center of the spin column membrane
12. Incubate for 1 minute at room temperature to elute the extracted DNA
13. Collect the DNA by centrifuging for 1 minute at 8,000 RPM

Cetyltrimethylammonium Bromide (CTAB) Extraction

1. Add 500 µl of CTAB Extraction Buffer to the tick
2. Place the solution in a 60°C water bath for 30 minutes
3. Centrifuge the solution at 10,000 RPM for 5 minutes and transfer supernatant to a clean tube
4. Add five µl of RNase A (10 mg/ml) to the supernatant
5. Mix the sample by vortexing for 5 seconds
6. Incubate at room temperature for 15 minutes
7. Add 350 µl of 100% isopropanol to the sample
8. Vortex for five seconds and incubate at -20°C for 15 minutes
9. Transfer sample to a spin column and centrifuge at 8,000 RPM for 4 minutes
10. Discard the flow through and add 200 µl of ice cold 70% ethanol to the spin column
11. Centrifuge at 8,000 RPM for 2 minutes, discard the flow through
12. Add 200 µl of ice cold 70% ethanol to the spin column
13. Centrifuge at 8,000 RPM for 2 minutes, discard the flow through
14. Centrifuge for 5 minutes at 14,000 RPM to remove any residual ethanol
15. Transfer the spin columns to a clean microcentrifuge tube
16. Add 100 µl of TE buffer to elute the DNA
17. Incubate at room temperature for 5 minutes
18. Centrifuge at 8,000 RPM for one minute
