# Supplementary material for: Comparison of DNA Extraction and Amplification Techniques for Use with Engorged Hard-Bodied Ticks
Source: Microorganisms. 2022 Jun 20;10(6):1254. doi: 10.3390/microorganisms10061254 (PMC9228219; doi:10.3390/microorganisms10061254)

Gel A
1.CTAB-WB-1
2.CTAB-WB-2
3.CTAB-WB-3
4.CTAB-WB-4
5.CTAB-WB-5
6.CTAB-WB-6
7.CTAB-WB-7
8.CTAB-WB-8
9.CTAB-WB-9
10.CTAB-WB-10
11.CTAB-WB-11
12.Not used in analysis
13.CTAB-AC-12
14.CTAB-AC-13
15.CTAB-AC-14
16.CTAB-AC-15
17.CTAB-AC-16
18.CTAB-AC-17
19.CTAB-AC-18
All samples were amplified using AmpliTaq

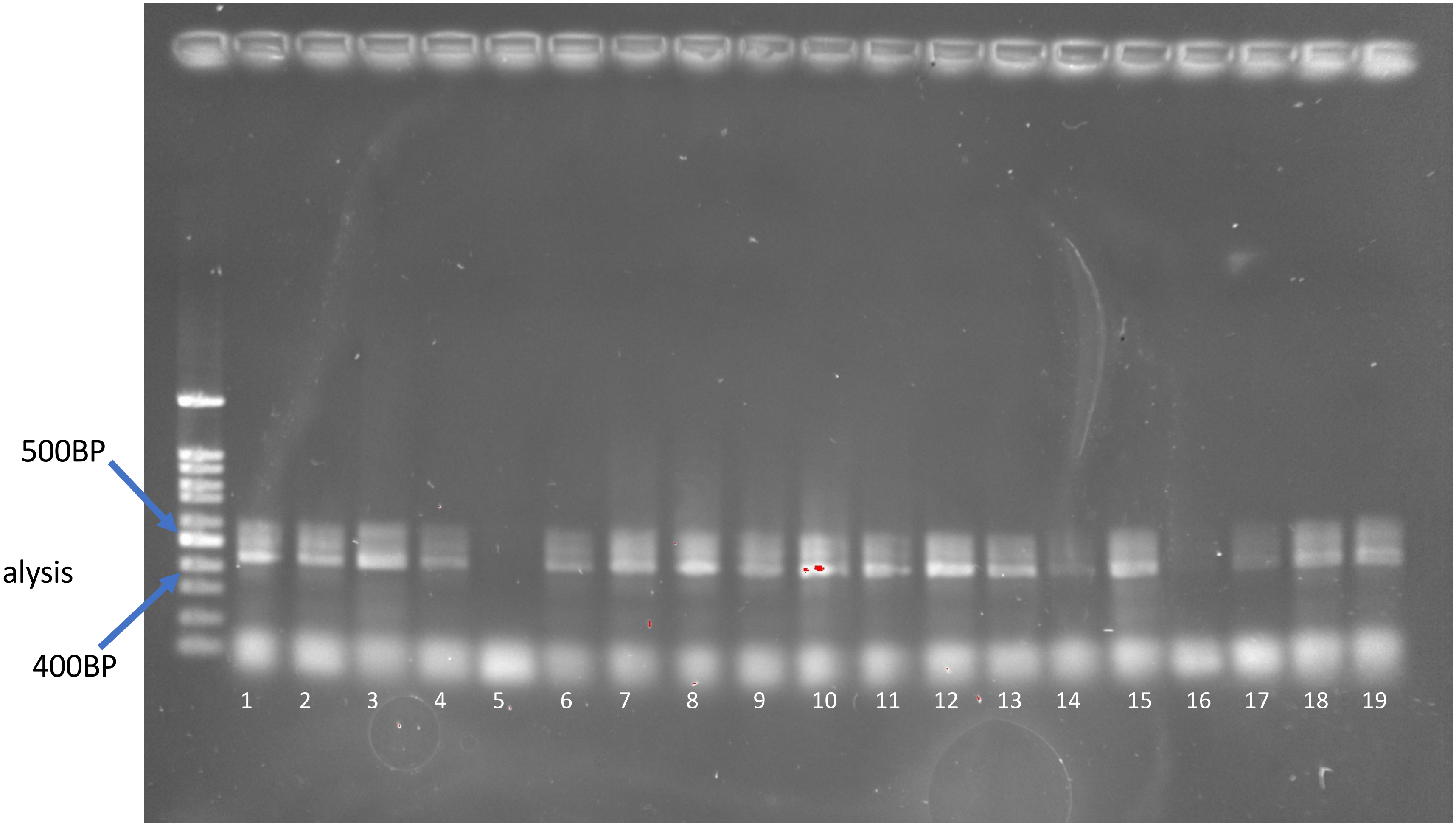

Gel B
1. CTAB-AC-19
2. CTAB-AC-20
3. CTAB-AC-21
4. CTAB-AC-22
5. Not used in analysis
6. Not used in analysis
7. Not used in analysis
8. Not used in analysis
9. Not used in analysis
10. Not used in analysis
11. Not used in analysis
12. Not used in analysis
13. Not used in analysis
14. Not used in analysis
15. Not used in analysis
16. Not used in analysis
17. Not used in analysis
18. Not used in analysis
19. Not used in analysis

All samples were amplified using AmpliTaq

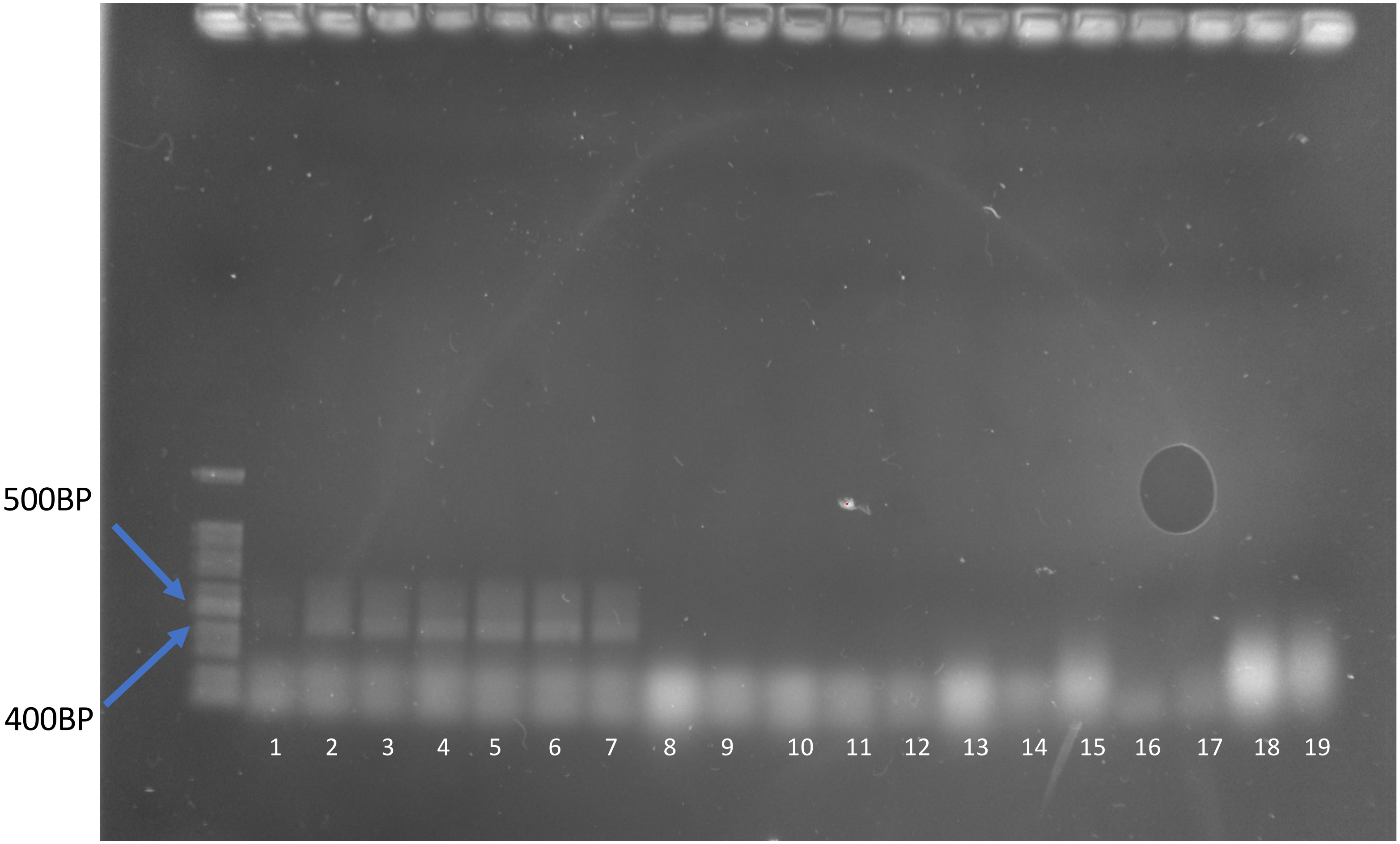

Gel C

1. Ph-Cl-WB-1
2. Ph-Cl-WB-2
3. Ph-Cl-WB-3
4. Ph-Cl-WB-4
5. Ph-Cl-WB-5
6. Ph-Cl-WB-6
7. Ph-Cl-WB-7
8. Ph-Cl-WB-8
9. Ph-Cl-WB-9
10. Ph-Cl-WB-10
11. Ph-Cl-WB-11
12. Ph-Cl-AC-12
13. Ph-Cl-AC-13
14. Ph-Cl-AC-14
15. Ph-Cl-AC-15
16. Ph-Cl-AC-16
17. Ph-Cl-AC-17
18. Ph-Cl-AC-18
19. Ph-Cl-AC-19
20. Ph-Cl-AC-20
21. Ph-Cl-AC-21
22. Ph-Cl-AC-22
23. Not used in analysis
24. Not used in analysis
25. Positive Control
26. Negative Control

All samples were amplified using AmpliTaq

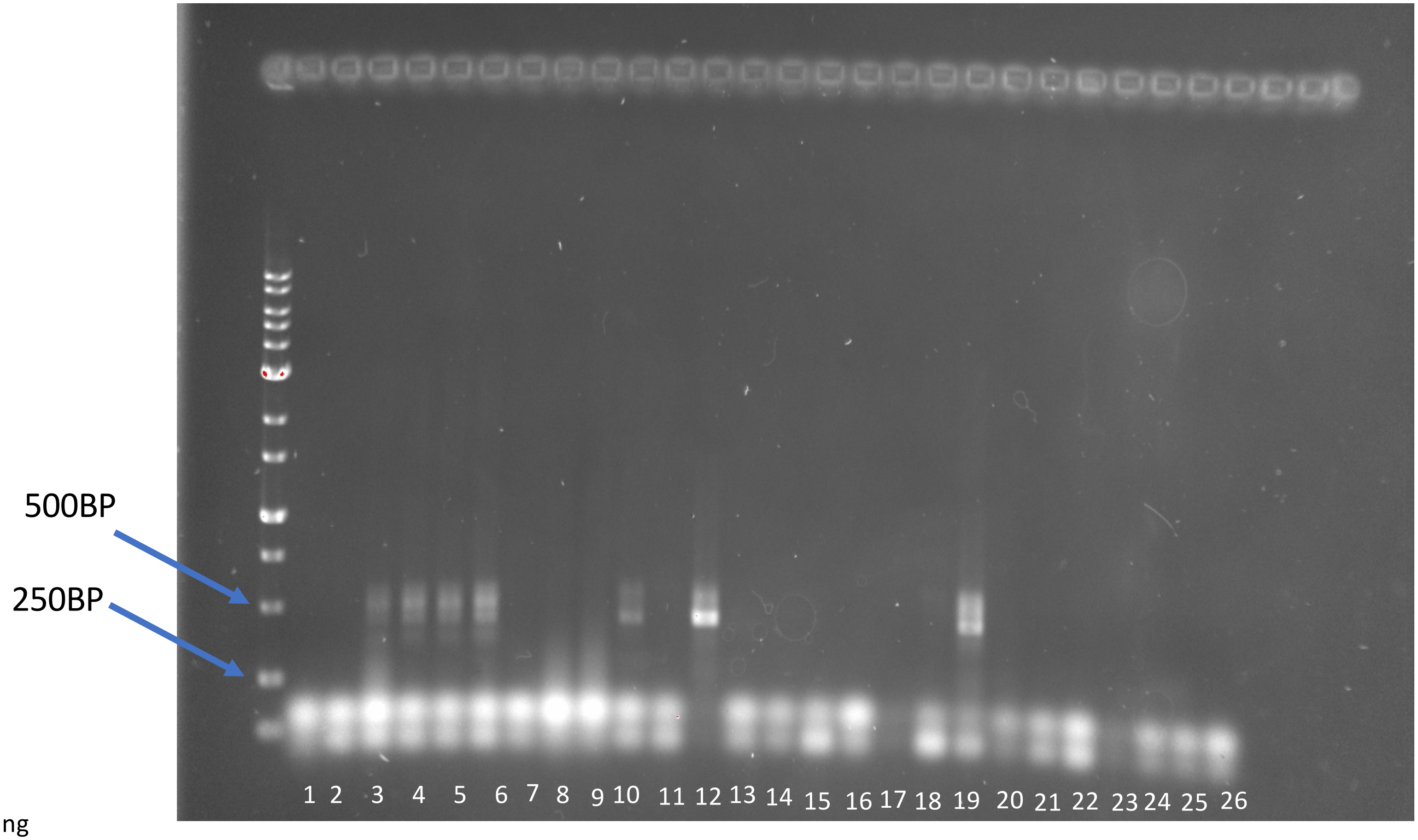

Gel D
1. Qiagen-WB-1
2. Not used in analysis
3. Qiagen-WB-2
4. Qiagen-WB-3
5. Qiagen-WB-4
6. Qiagen-WB-5
7. Qiagen-WB-6
8. Qiagen-WB-7
9. Qiagen-WB-8
10. Qiagen-WB-9
11. Qiagen-WB-10
12. Qiagen-WB-11
13.Not used in analysis
14. Qiagen-AC-12
15. Qiagen-AC-13
16. Qiagen-AC-14
17. Qiagen-AC-15
18. Qiagen-AC-16
19.Qiagen-AC-17
All samples were amplified with AmpliTaq

500BP

400BP

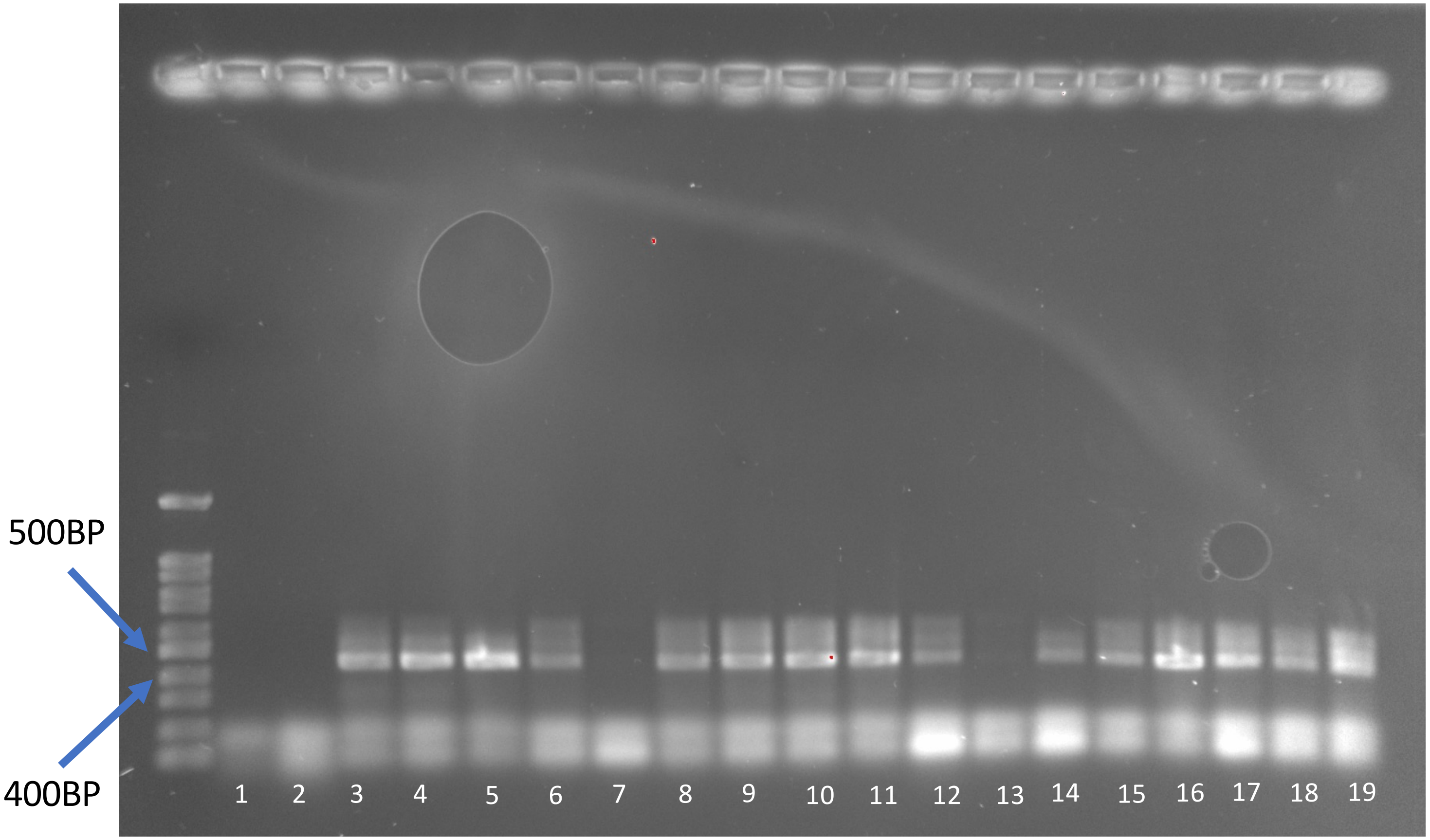

Gel E
1. Qiagen-AC-18
2. Qiagen-AC-19
3. Qiagen-AC-20
4. Qiagen-AC-21
5. Qiagen-AC-22
6. Negative Control
7. Qiagen-AC-18
8. Qiagen-AC-19
9. Qiagen-AC-20
10. Qiagen-AC-21
11. Qiagen-AC-22
12.Negative Control
Samples 1-5 were amplified using GoTaq
Samples 7-11 were amplified using Ampligold

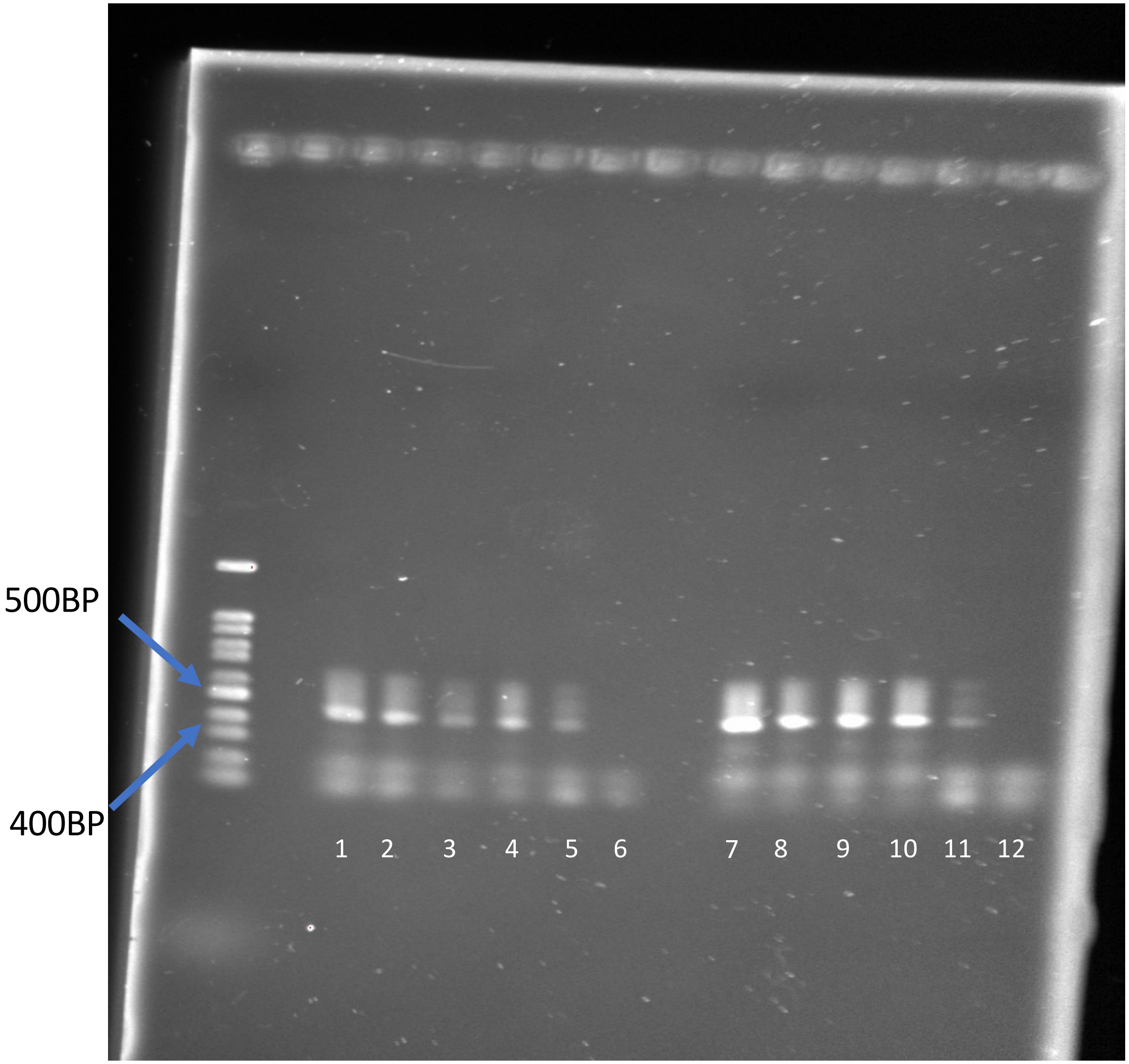

Gel F
1. Qiagen-WB-1
2. Qiagen-WB-2
3. Qiagen-WB-3
4. Qiagen-WB-4
5. Qiagen-WB-5
6. Qiagen-WB-6
7. Qiagen-WB-7
8. Qiagen-WB-8
9. Qiagen-WB-9
10. Qiagen-WB-10
11. Qiagen-WB-11
12. Qiagen-AC-12
13.Qiagen-AC-13
14. Qiagen-AC-14
15. Qiagen-AC-15
16. Qiagen-AC-16
17. Qiagen-AC-17
18. Positive Control
19. Negative Control
All samples were amplified with GoTaq

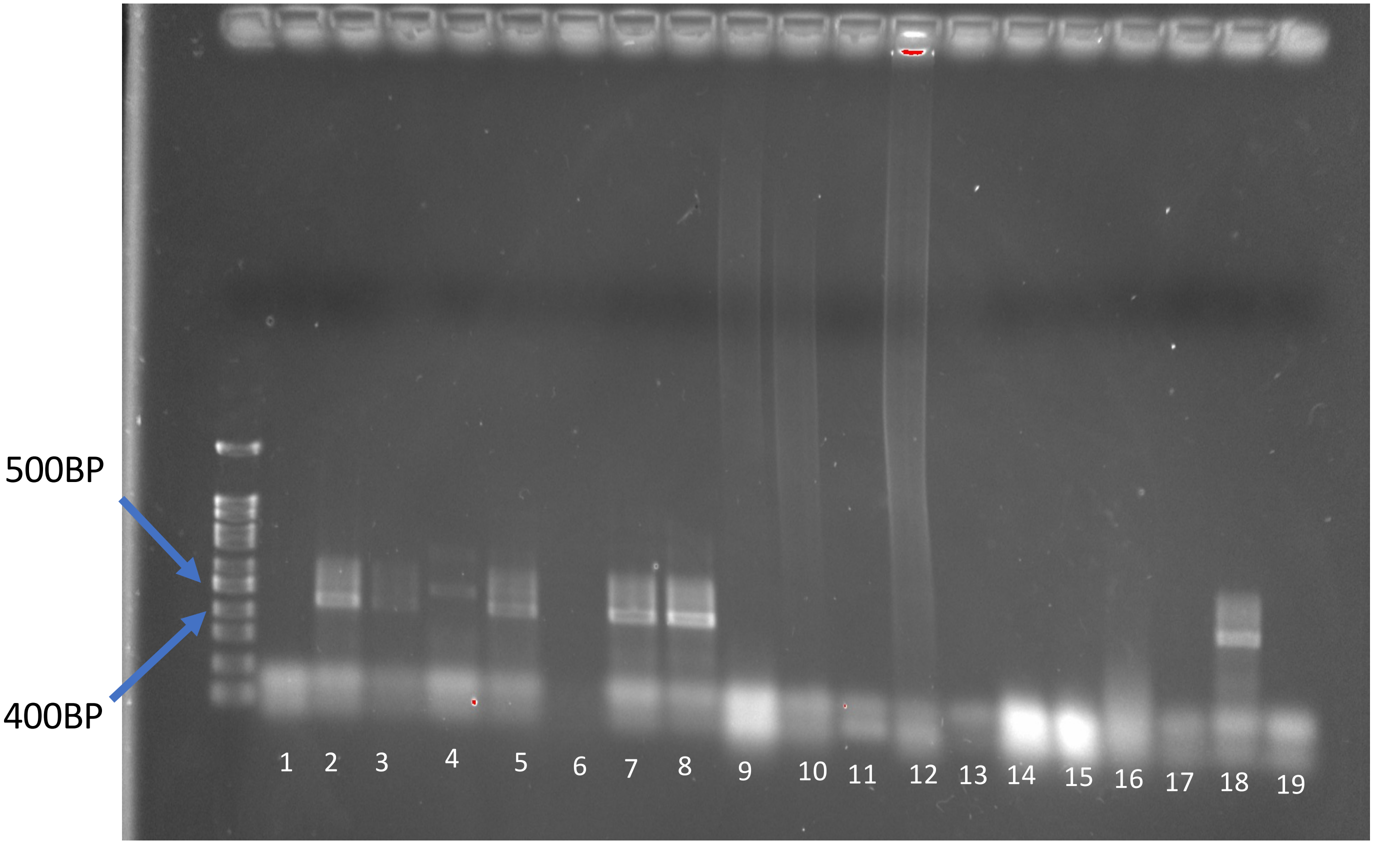

Gel G
1. CTAB-WB-1
2. CTAB-WB-2
3. CTAB-WB-3
4. CTAB-WB-4
5. CTAB-WB-5
6. CTAB-WB-6
7. CTAB-WB-7
8. CTAB-WB-8
9. CTAB-WB-9
10. CTAB-WB-10
11. CTAB-WB-11
12. CTAB-AC-12
13. CTAB-AC-13
14. CTAB-AC-14
15. CTAB-AC-15
16. CTAB-AC-16
17. CTAB-AC-17
18. CTAB-AC-18
19. CTAB-AC-19
All samples were amplified using GoTaq

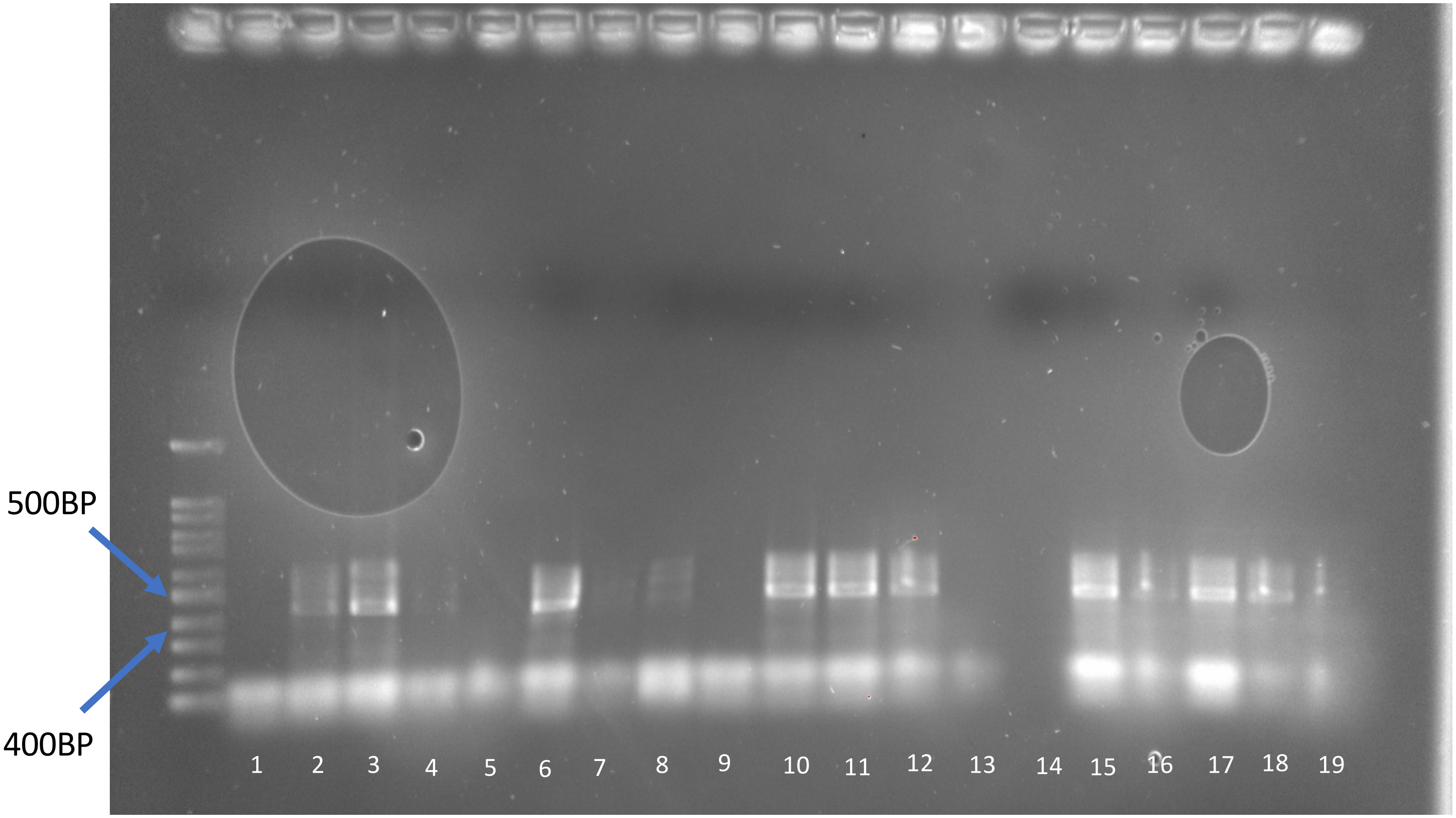

Gel H

1. Ph-Cl-WB-1
2. Ph-Cl-WB-2
3. Ph-Cl-WB-3
4. Ph-Cl-WB-4
5. Ph-Cl-WB-5
6. Ph-Cl-WB-6
7. Ph-Cl-WB-7
8. Ph-Cl-WB-8
9. Ph-Cl-WB-9
10. Ph-Cl-WB-10
11. Ph-Cl-WB-11
12. Ph-Cl-AC-12
13. Ph-Cl-AC-13
14. Ph-Cl-AC-14
15. Ph-Cl-AC-15
16. Ph-Cl-AC-16
17. Ph-Cl-AC-17
18. Ph-Cl-AC-18
19. Ph-Cl-AC-19
20. Ph-Cl-AC-20
21. Ph-Cl-AC-21
22. Ph-Cl-AC-22
23. Not used in analysis
24. Not used in analysis
25. Positive Control
26. Negative Control

All samples were amplified using GoTaq

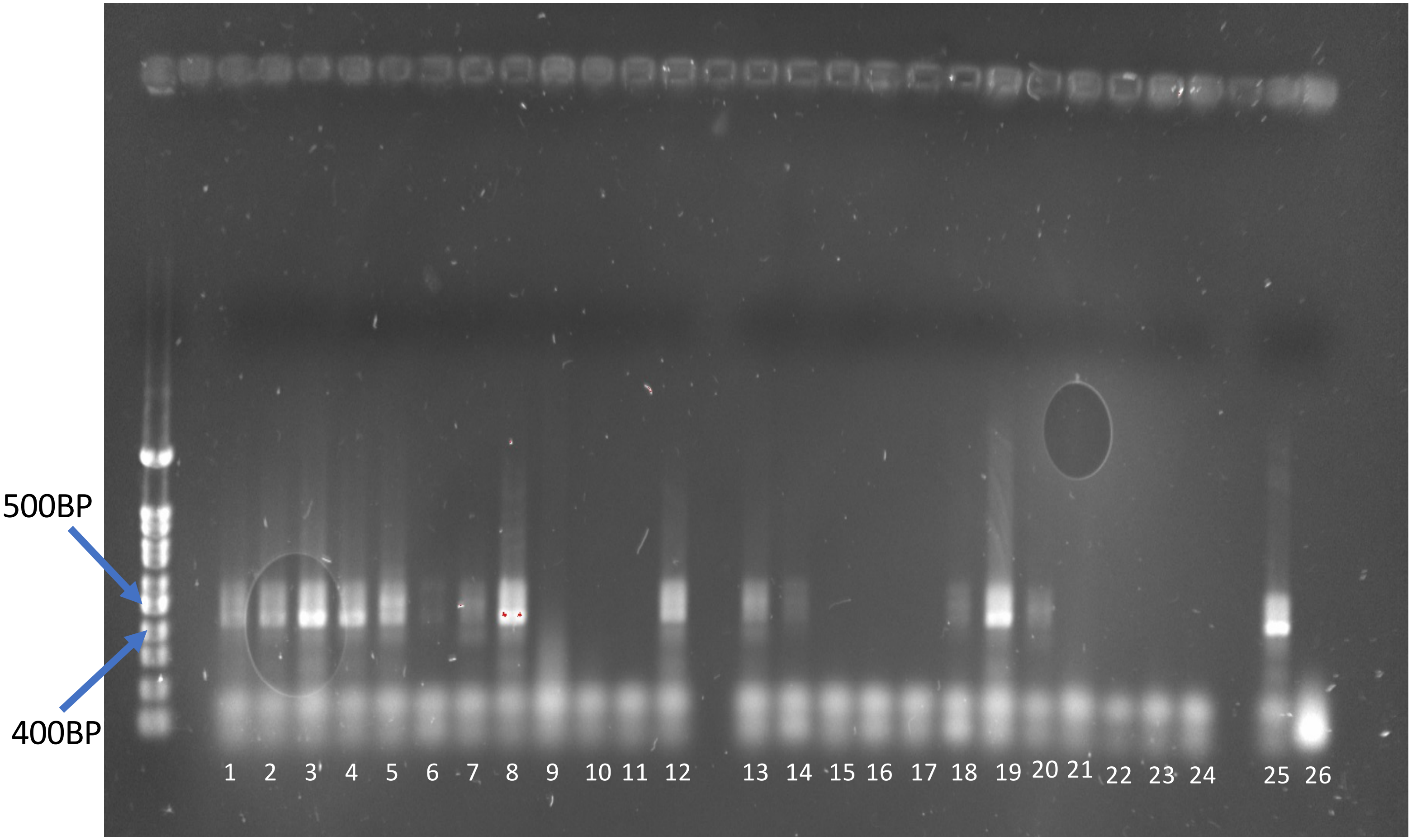

Gel I

1. Ph-Cl-WB-1
2. Ph-Cl-WB-2
3. Ph-Cl-WB-3
4. Ph-Cl-WB-4
5. Ph-Cl-WB-5
6. Ph-Cl-WB-6
7. Ph-Cl-WB-7
8. Ph-Cl-WB-8
9. Ph-Cl-WB-9
10. Ph-Cl-WB-10
11. Ph-Cl-WB-11
12. Ph-Cl-AC-12
13. Ph-Cl-AC-13
14. Ph-Cl-AC-14
15. Ph-Cl-AC-15
16. Ph-Cl-AC-16
17. Ph-Cl-AC-17
18. Ph-Cl-AC-18
19. Ph-Cl-AC-19
20. Ph-Cl-AC-20
21. Ph-Cl-AC-21
22. Ph-Cl-AC-22
23. Not used in analysis
24. Not used in analysis
25. Positive Control
26. Negative Control

All samples were amplified using Phusion Taq

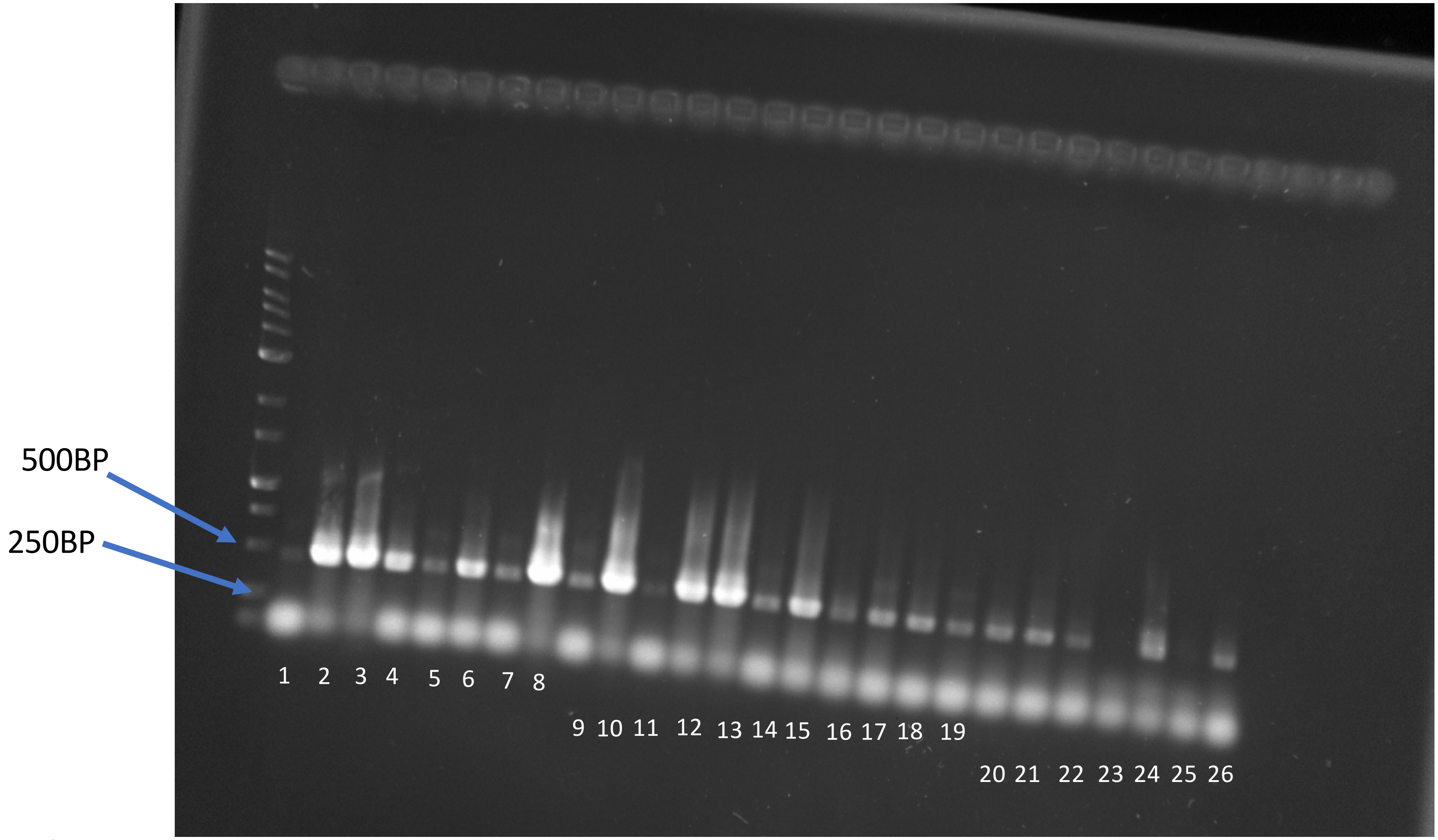

Gel J
1. Qiagen-WB-1
2. Qiagen-WB-2
3. Qiagen-WB-3
4. Qiagen-WB-4
5. Qiagen-WB-5
6. Qiagen-WB-6
7. Qiagen-WB-7
8. Qiagen-WB-8
9. Qiagen-WB-9
10. Qiagen-WB-10
11. Qiagen-WB-11
12. Qiagen-AC-12
13. Qiagen-AC-13
14. Qiagen-AC-14
15. Qiagen-AC-15
16. Qiagen-AC-16
17. Qiagen-AC-17
18. Qiagen-AC-18
19.Qiagen-AC-19
All samples were amplified with Phusion Taq

500BP

400BP

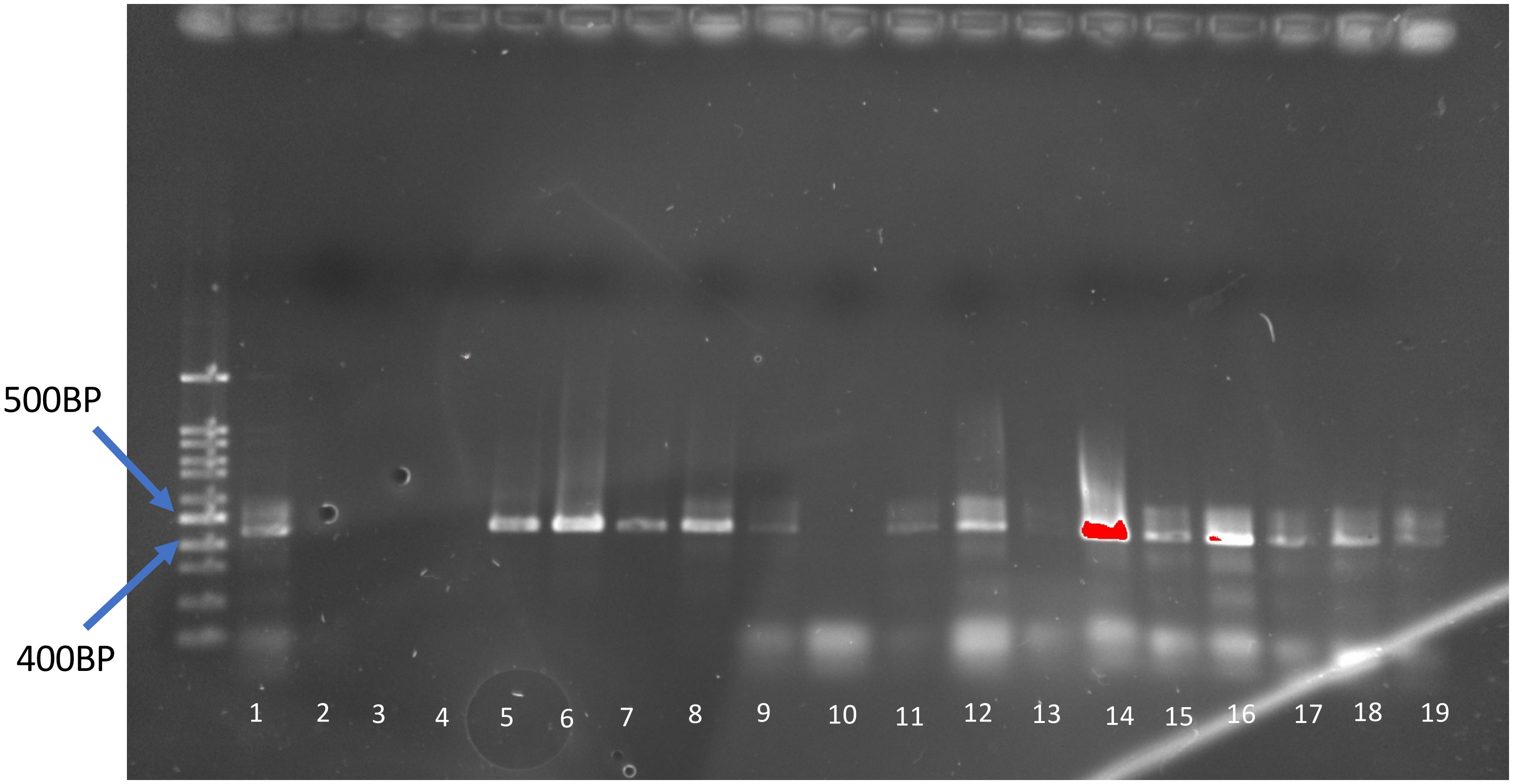

Gel K
1. CTAB-WB-1
2. CTAB-WB-2
3. CTAB-WB-3
4. CTAB-WB-4
5. CTAB-WB-5
6. CTAB-WB-6
7. CTAB-WB-7
8. CTAB-WB-8
9. CTAB-WB-9
10. CTAB-WB-10
11. CTAB-WB-11
12. CTAB-AC-12
13. CTAB-AC-13
14. CTAB-AC-14
15. CTAB-AC-15
16. CTAB-AC-16
17. CTAB-AC-17
18. CTAB-AC-18
19. CTAB-AC-19
20. CTAB-AC-20
21. CTAB-AC-21
22. CTAB-AC-22
23. Negative Control
All samples were amplified using Phusion Taq

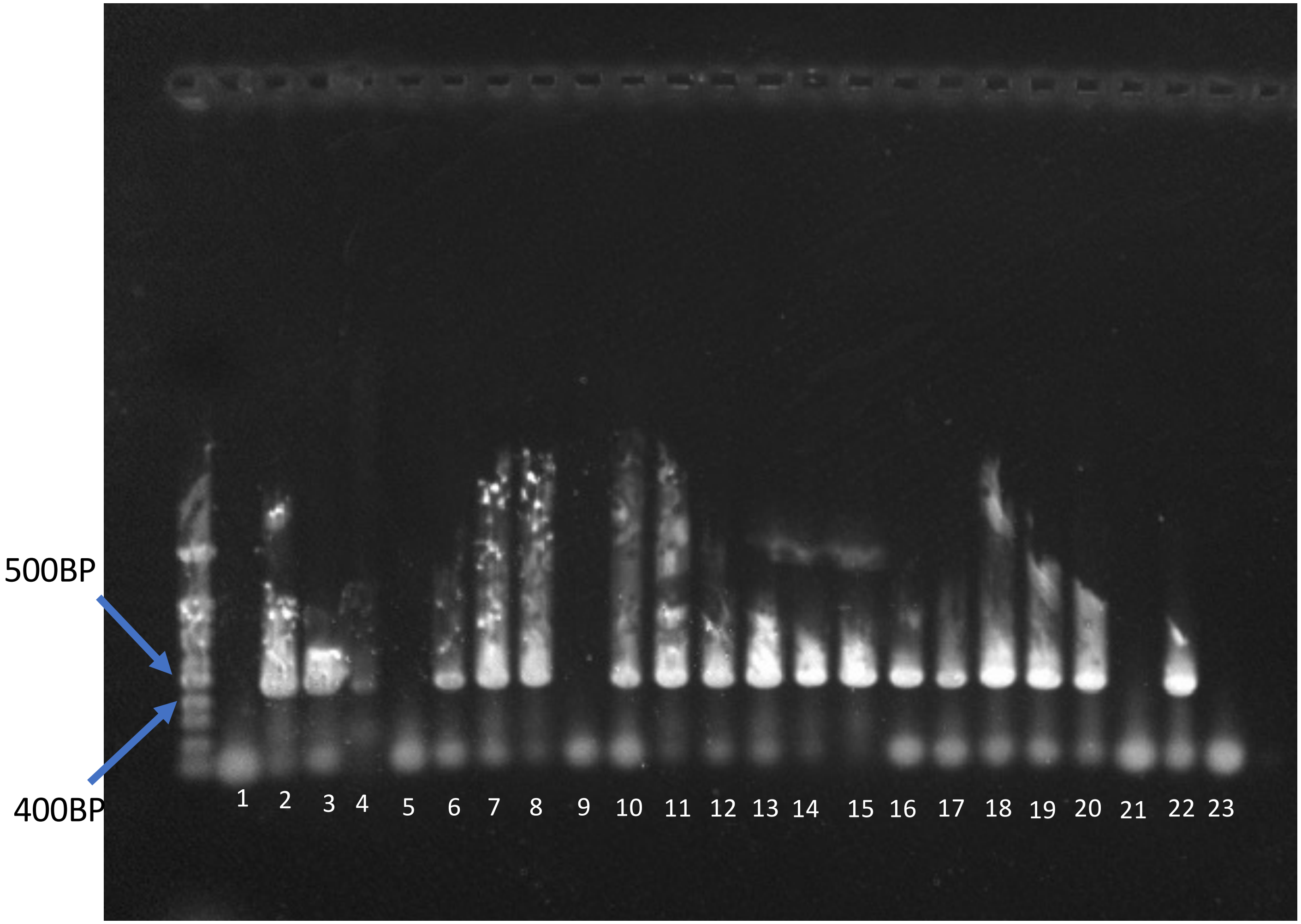

Gel L

1. Ph-Cl-WB-1
2. Ph-Cl-WB-2
3. Ph-Cl-WB-3
4. Ph-Cl-WB-4
5. Ph-Cl-WB-5
6. Ph-Cl-WB-6
7. Ph-Cl-WB-7
8. Ph-Cl-WB-8
9. Ph-Cl-WB-9
10. Ph-Cl-WB-10
11. Ph-Cl-WB-11
12. Ph-Cl-AC-12
13. Ph-Cl-AC-13
14. Ph-Cl-AC-14
15. Ph-Cl-AC-15
16. Ph-Cl-AC-16
17. Ph-Cl-AC-17
18. Ph-Cl-AC-18
19. Ph-Cl-AC-19
20. Ph-Cl-AC-20
21. Ph-Cl-AC-21
22. Ph-Cl-AC-22
23. Not used in analysis
24. Not used in analysis
25. Positive Control
26. Negative Control

All samples were amplified using HemoKlen Taq

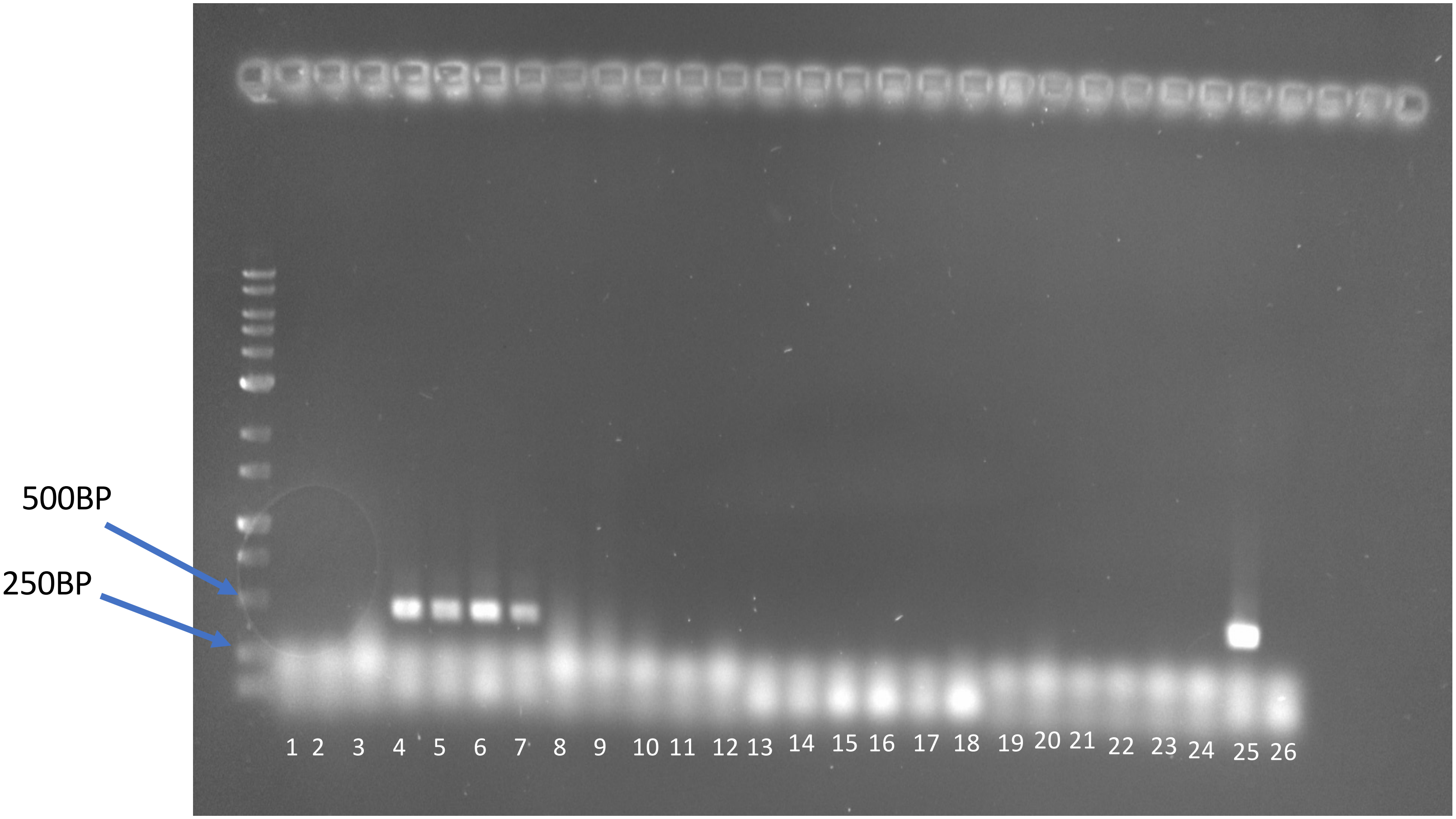

Gel M

1. Qiagen-WB-1
2. Qiagen-WB-2
3. Qiagen-WB-3
4. Qiagen-WB-4
5. Qiagen-WB-5
6. Qiagen-WB-6
7. Qiagen-WB-7
8. Qiagen-WB-8
9. Qiagen-WB-9
10. Qiagen-WB-10
11. Qiagen-WB-11
12. Qiagen-AC-12
13. Qiagen-AC-13
14. Qiagen-AC-14
15. Qiagen-AC-15
16. Qiagen-AC-16
17. Qiagen-AC-17
18. Qiagen-AC-18
19. Qiagen-AC-19
20. Qiagen-AC-20
21. Qiagen-AC-21
22. Qiagen-AC-22
23. Not used in analysis
24. Not used in analysis
25. Negative Control
26. Positive Control

All samples were amplified using HemoKlen

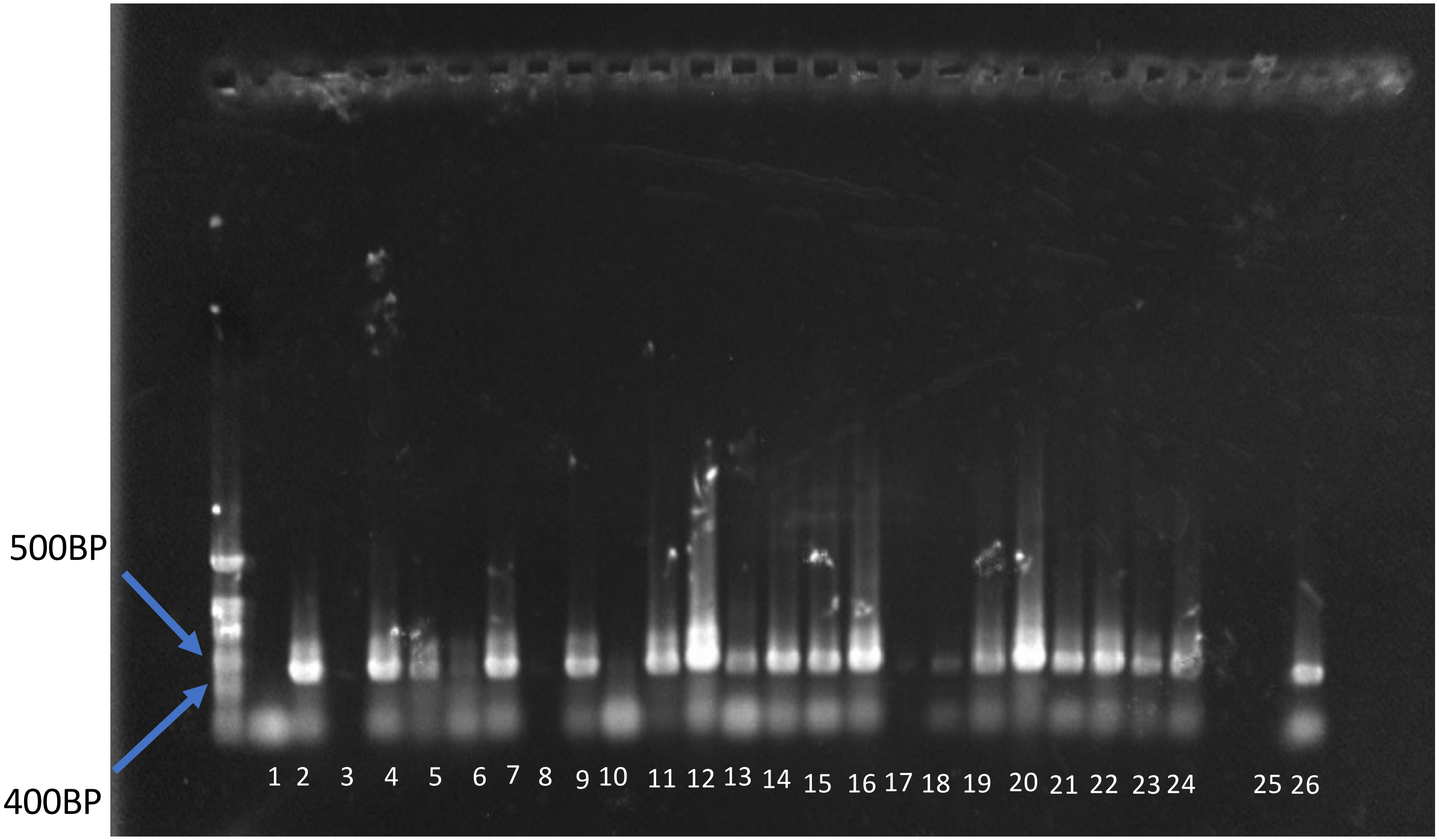

Gel N
1. Qiagen-WB-20
2. Qiagen-WB-21
3. Qiagen-WB-22
4. Not used in analysis
5. Not used in analysis
6. Not used in analysis
7. Not used in analysis
8. Not used in analysis
9. Not used in analysis
10. Not used in analysis
11. Not used in analysis
12. Not used in analysis
13. Positive Control
All samples were amplified using Phusion Taq

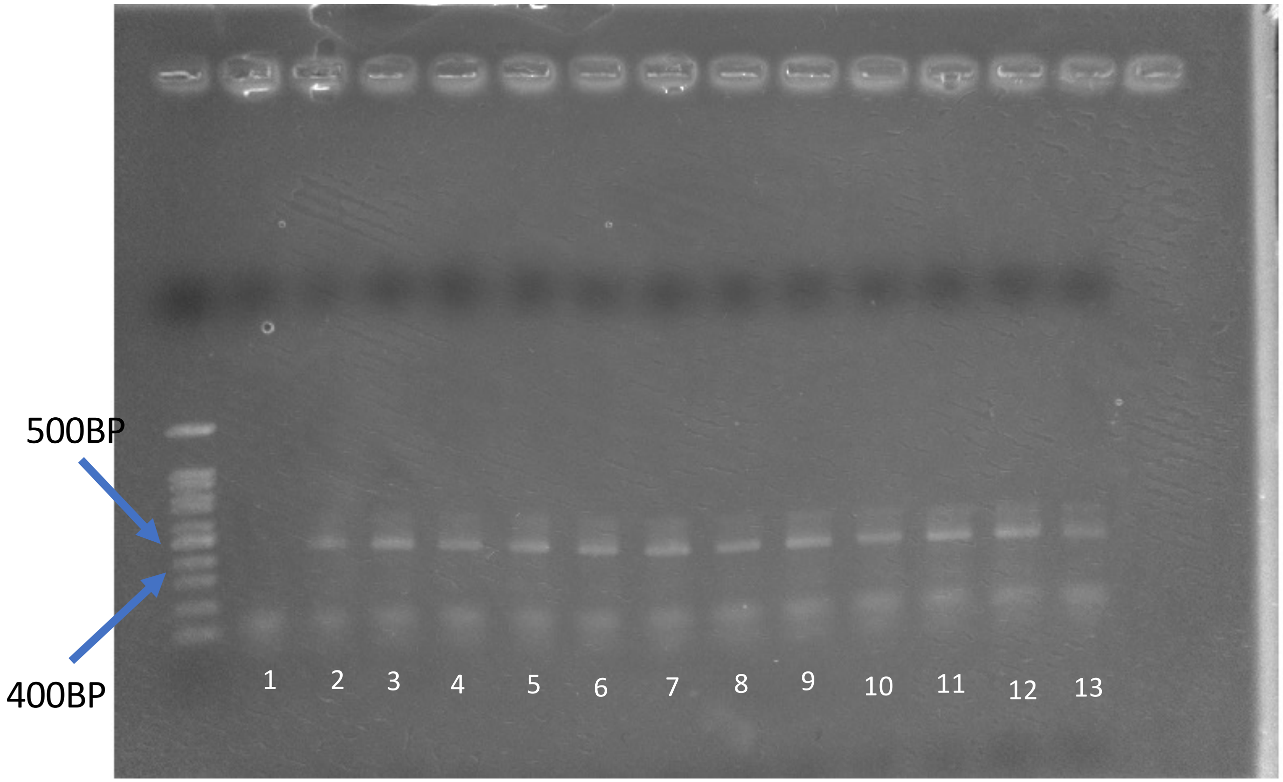

Gel O

1. CTAB-WB-1
2. CTAB-WB-2
3. CTAB-WB-3
4. CTAB-WB-4
5. CTAB-WB-5
6. CTAB-WB-6
7. CTAB-WB-7
8. CTAB-WB-8
9. CTAB-WB-9
10. CTAB-WB-10
11. CTAB-WB-11
12. CTAB-AC-12
13. CTAB-AC-13
14. CTAB-AC-14
15. CTAB-AC-15
16. CTAB-AC-16
17. CTAB-AC-17
18. CTAB-AC-18
19. CTAB-AC-19
20. CTAB-AC-20
21. CTAB-AC-21
22. CTAB-AC-22
23. Positive Control

All samples were amplified using Hemoklen Taq

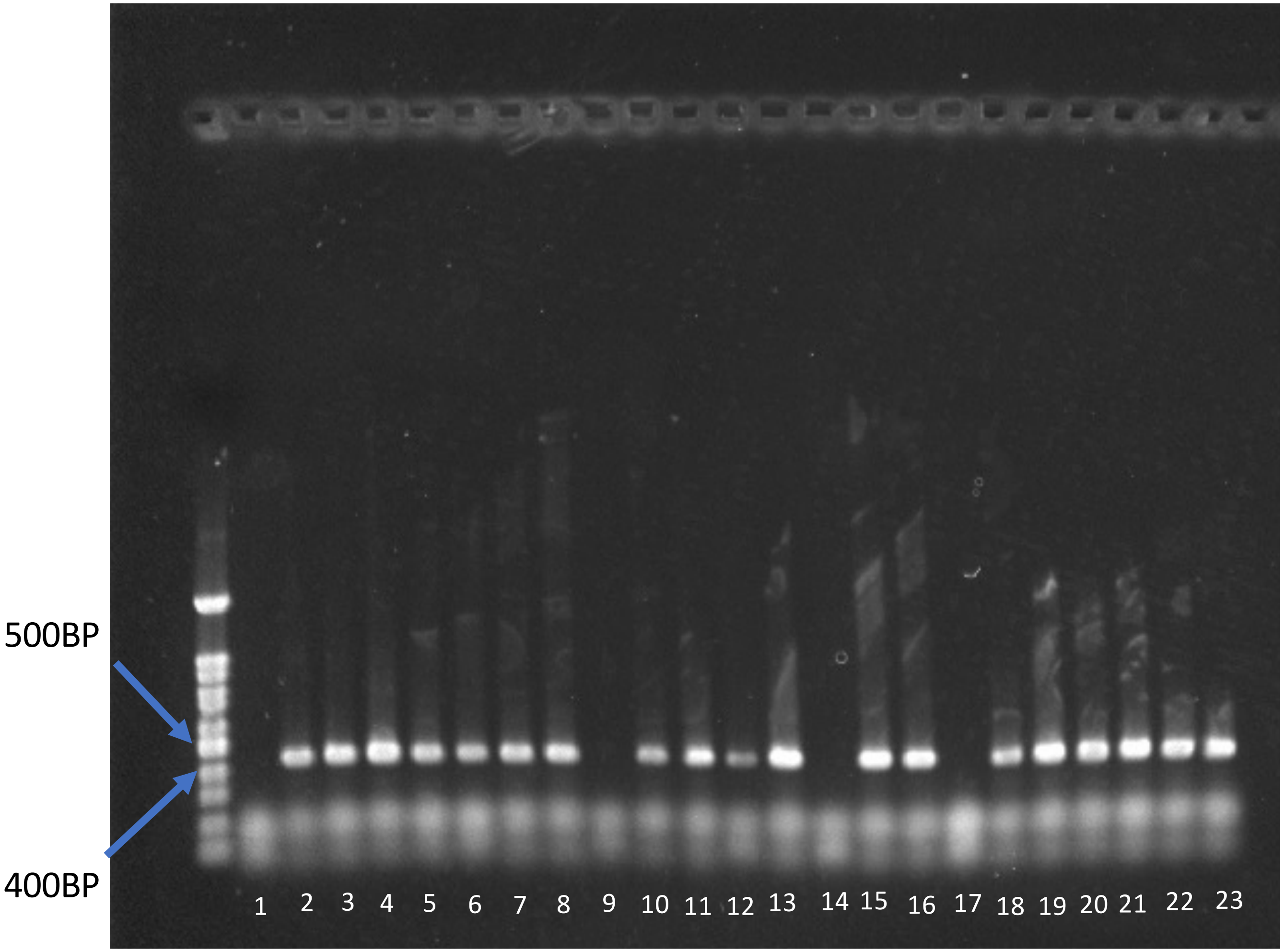

Supplement: Supplementary file 1 [file microorganisms-10-01254-s001.zip › Supplemental Materials File S2_Labeled Gel Images.pdf]
